# Data integration and synthesis for pandemic and epidemic intelligence

**DOI:** 10.1186/s12919-025-00321-9

**Published:** 2025-04-23

**Authors:** Barbara Tornimbene, Zoila Beatriz Leiva Rioja, Manoel Barral-Netto, Carlos Castillo-Salgado, Irena Djordjevic, Moritz Kraemer, Martina McMenamin, Oliver Morgan

**Affiliations:** 1 World Health Organization Hub for Pandemic and Epidemic Intelligence, Berlin, Germany; 2 CPC Analytics, Berlin, Germany; 3 Oswaldo Cruz Foundation (FIOCRUZ), Salvador, Bahia Brasil; 4 Johns Hopkins University (JHU), Baltimore, MD USA; 5 World Health Organization Head Quarter, Geneva, Switzerland; 6 University of Oxford, Oxford, UK

**Keywords:** Data integration, Pandemic intelligence, Epidemic preparedness, Public health surveillance, Data interoperability, Health informatics, Real-time data sharing, Global health systems

## Abstract

The COVID-19 pandemic highlighted substantial obstacles in real-time data generation and management needed for clinical research and epidemiological analysis. Three years after the pandemic, reflection on the difficulties of data integration offers potential to improve emergency preparedness. The fourth session of the WHO Pandemic and Epidemic Intelligence Forum sought to report the experiences of key global institutions in data integration and synthesis, with the aim of identifying solutions for effective integration. Data integration, defined as the combination of heterogeneous sources into a cohesive system, allows for combining epidemiological data with contextual elements such as socioeconomic determinants to create a more complete picture of disease patterns. The approach is critical for predicting outbreaks, determining disease burden, and evaluating interventions. The use of contextual information improves real-time intelligence and risk assessments, allowing for faster outbreak responses. This report captures the growing acknowledgment of data integration importance in boosting public health intelligence and readiness and show examples of how global institutions are strengthening initiatives to respond to this need. However, obstacles persist, including interoperability, data standardization, and ethical considerations. The success of future data integration efforts will be determined by the development of a common technical and legal framework, the promotion of global collaboration, and the protection of sensitive data. Ultimately, effective data integration can potentially transform public health intelligence and our way to successfully respond to future pandemics.

## Introduction

The COVID-19 pandemic resulted in a demand for real-time data collection that many healthcare systems struggled to manage [1]. These data were needed for clinical research, epidemiological analysis, mathematical modelling, and genomic analysis, among other uses. Three years after the pandemic was declared, it is possible to begin reflecting on the data challenges that arose and how health organizations can be more proactive in facilitating data integration to enhance future infectious disease emergency response capabilities [2]. In the context of the COVID-19 pandemic, timely consolidation of volumes of data from disparate sources, including contextual information, and ensuring their avaialbility can provide the public health workforce and policy makers with actionable insights that can help minimize the impact of disease outbreaks.

During the fourth World Health Organization (WHO) Pandemic and Epidemic Intelligence Innovation Forum [3], convened on 15th September 2022, colleagues from Johns Hopkins University (JHU) Bloomberg School of Public Health [4], Oswaldo Cruz Foundation (Fiocruz) [5], University of Oxford [6], and the WHO Global COVID-19 Incident Management Team shared their experiences of data integration and synthesis, identifying some prospects for effective integration of large amounts of data. The meeting provided an opportunity to discuss the benefits of data integration and which types of data should be considered for this process. They discussed their current work, together with the necessary digital infrastructure and challenges met in data synthesis. Finally, the importance of open collaboration and knowledge sharing in the field of data integration was highlighted.

### Data integration for public health

Data integration and synthesis can be defined as the combination of different data sources with the aim of providing a unified view of data [7]. The need for data integration grows as the volume and the need to share existing data increase. The data being integrated are received from diverse database systems and transformed into a single coherent database schema. The term "schema" refers to the organization of data as a blueprint of how the database is constructed. Integration begins with the ingestion process and includes steps such as cleaning, data mapping, and transformation. Data integration ultimately enables analytics tools to produce effective and actionable intelligence and it encourages collaboration between internal as well as external users [8].

In the context of public health, one example of data integration can be seen as the combination of the information related to all the factors that play a role in disease occurrence, prevention, and control. It can start from merging strictly epidemiological data on disease occurrence (prevalence, incidence, mortality) and response (testing protocols, vaccine rollouts) with contextual factors, including social and environmental determinants of health. For example, it can be the integration of patient data with health-seeking behaviour data, or of syndromic data with medication sales, poison control data, absenteeism, and laboratory tests and results [9]. In an even more holistic approach, data integration in the public health context needs the creation of repositories of contextual information that allow for a better understanding of how health events affect people in their communities. Contextual information can include any information that improves interpretation, such as the socio-economic composition of affected communities, travel patterns, housing and environment, conflict, animal reservoir populations, health systems capacities, deployment of medical countermeasures, and social factors, such as public sentiment about disease-control measures, among many others [10]. All together this extra information allows for better assessment of risks, a clearer understanding of disease drivers and impacts in different settings, and a stronger basis for public health decision making.

### The rising wave of data integration

Better containment of emerging infectious disease outbreaks, such as the 2014 Ebola crisis and the COVID-19 pandemic, require rapidly available insights into the epidemiological characteristics of the outbreak, from transmission potential to their natural history. This implies a rapid scale-up of testing and pathogen genomic sequencing (where relevant), a fast assessment of clinical impact, and open sharing of early findings. As the outbreak grows, forecasting of disease dynamics, estimation of potential burden, and evaluation of interventions become key considerations.

Yet, the availability of information is usually very limited, and the collection process of these data, including case definitions is often unknown making obtaining robust estimates of transmission routes, infectiousness of the pathogen, or disease severity extremely challenging. Furthermore, variation in available information inhibits the ability to perform the standardization of risk assessments across multiple settings. Nevertheless, scientists are often required to make inferences based on these early and incomplete data to inform policy decisions. A better understanding of the context where diseases occur can help deliver more robust information to support core public health functions and decision-making processes, as well as to provide insights for rapid disease control.

For this reason, during the last years, more effort has been spent developing effective approaches to integrate and synthesize data from different sources to generate contextual information. Some of the factors that can impact or determine bias in early estimates are health care seeking behaviour, populations accessibility to health centres and the protocol of testing, which are known to vary both between countries and within countries over time. In this regard, data integration offers access to complex datasets containing this information so that it can be used in early stages to better inform decision-makers, as well as the public. These large repositories of information allow for a better characterization of the settings in which the outbreak is occurring.

The contextualization of health events can help conduct better risk assessments. It also supports the strengthening of epidemic preparedness and early outbreak alert and risk prediction, by enriching real time intelligence. Most traditional public health surveillance systems rely on stable case ascertainment over time and a robust baseline against which changes can be assessed. An outbreak is classically defined by changes in case incidence in the context of person, place, or time. However, this paradigm is often not sufficient for the rapid detection of emerging infectious diseases when early case numbers are small, where no historic baseline has been established, or where laboratory confirmation of cases is uncertain [10]. In this case, data integration helps to increase prediction performance by allowing consideration of the diverse determinants of health that impact the risk of disease occurrence, and it also helps identify biases in data and adjusting them. Moreover, bringing together local and regional knowledge has proven to be extremely useful to assess outbreak situations as professionals who are representative of communities (in terms of gender, race, ethnicity, geographical origin, class, etc.) have a better understanding of their needs and dynamics.

### How some global institutions are moving towards data integration

The JHU Bloomberg School of Public Health [4]recognized the importance of characterizing information, indicators, and data to allow for synthesis that could provide rapid and standardized assessments of global health emergencies. They specifically recognised the role of identifying the population profiles, both in the socio-economic and health realms, and the response capacity of health systems, in delivering public health situation analysis, measuring inequalities, carrying out needs assessments, and setting priorities. Within this scope, they have worked with the Pan American Health Organization (PAHO) to select core indicators (*n* = 116) that, once integrated, could be used by countries in different domains, from demographics, socioeconomic status, morbidity, mortality, and the capacity of the health system, as framework for rapid and comparable national and regional health assessments.

In an even wider perspective, the University of Oxford [6] has started developing a repository where health data come together from multiple countries with the associated metadata. This effort grew into a global consortium. Their data repository includes basic demographics of countries' populations, such as age, sex, and location that can be accessed and shared globally by many partners. Where available, they track information on travel history, people's health seeking behaviour, pre-existing conditions, infections reproduction numbers, and community transmission during an outbreak to better understand and model the burden of disease. They also integrate information with the Global Health Data Exchange (GHDx) [11], which provides sizable contextual data, as environmental or population level data. The main goal is to share some of this information in an open-source setting to permit the generation of more robust estimates for the public, but also for policymakers’ decision-making.

Following similar steps, Fiocruz [5] instituted The Centre for Data and Knowledge Integration for Health (CIDACS) [12] to link health and social data in Brazil and conduct studies and research based on interdisciplinarity. CIDAS created an ecosystem for data connectivity and it pulls together large databases from many of the ministries in Brazil, from which it is possible to generate a cohort that includes 130 million Brazilians [13]The component of early epidemic intelligence was developed in response to the pandemic: CIDACS Alert-early System of Outbreaks with Pandemic potential (AESOP) [14]. These databases contain information not only on health parameters, like mortality or hospitalization but also education and income. The goal of AESOP is to anticipate outbreak detection through merging information on several aspects of health with databases storing information on drug sales, environmental characteristics, bioclimatic effects, and other social determinants of health, and ultimately link it with components of advanced molecular pathogen characterization. Additionally, it aims to model outbreak dispersion thus providing the basis for rational containment strategies of known and unknown pathogens.

For example, in the surveillance of respiratory syndromes, a stepwise approach is planned to monitor respiratory syndromes which combines data from WHO Epidemic Intelligence from Open Sources (EIOS) initiative [15], press and social media with syndromic detection of excess cases of respiratory diseases in primary health care data and the sales of drugs used for respiratory infections. If the second level alert is triggered, field investigation and collection of clinical samples are initiated and simultaneously modelling based on social education and economic data, combined in the Brazilian Deprivation Index to evaluate health inequalities. If a large or fast dispersion is expected or if an unexpected etiological agent is confirmed, the third level alert is triggered, involving communication to federal level authorities and the application of IHR guidelines. A large group of bioclimatologists, geographers, and engineers from Federal University of Rio de Janeiro is also part of AESOP.

The WHO Global COVID-19 Incident Management Team developed a mixed-methods Global Alert System tool [16]that enables early identification of high-risk contexts to inform early COVID-19 response. The system aims to determine a risk of health systems becoming overwhelmed within each country using a three-stage process. The first stage is an automated algorithm which performs an epidemiological dynamics assessment to produce a prediction of mortality in the next five weeks, standardized by adjusting for underreporting, producing an initial alert level for each country. In the second stage, qualitative assessments of indicators using additional data sources, such as reported hospital and Intense Care Unit (ICU) occupancy and reports of concurrent outbreaks or conflict, are used to either upgrade or downgrade the initial alert level. Uncertainty around predictions and the quality of data sources is integrated throughout both stages to produce the final alert level. In the final stage, a weekly operational watchlist is produced to inform response activities and to prioritize operational and technical support. By merging epidemiological data on COVID-19 with a multifactorial context assessment, the tool facilitates the identification of those settings for which immediate actions (e.g., technical advice and assistance, operational support, advocacy, or rapid funding) may help mitigate the impact of a surge in COVID-19 morbidity or mortality.

### Challenges/relevant considerations for an effective data integration

There are certain elements which are critical for the success of data integration efforts. Interoperability and data integration require a common technical, legal, and organisational framework. In this regard, The University of Oxford’s project Global.Health [17] is encouraging the integration of technologies and the use of common digital tools and transformers. For example, they have engaged with the GORGAS Institute in Panama [18] and Stellenbosch University in South Africa [19], to promote locally based technology for those contexts where data are not shared internationally or are shared just in aggregate form. They are also working with the International Severe Acute Respiratory and emerging Infection Consortium (ISARIC) [20] to accelerate the use of data technologies in the context of clinical data integration.

The complexity of data integration derives from various factors such as data model structures, data formats, and data precision. There is often a lack of homogeneity in the merged data, not only in terms of organization, but also in terms of quality, as well as precision or representativeness. To address issue with data structure harmonization, the University of Oxford has built an infrastructure that codes data they receive from multiple datasets into a single schema [21]. To help define their schema, the Oxford team collaborates with health agencies and research laboratories, including the UK Health Security Agency and WHO, so that data can be more easily formatted and cleaned. This is an open process and once data are ready, they can be downloaded either through GitHub or the data portal. Likewise, Fiocruz is looking at different sources [22] to help with data harmonization, to control the vocabulary and taxonomy for a common data model. For example, one of these sources is the Observational Medical Outcomes Partnership (OMOP) Common Data Model (CDM) [23], which is an open community data standard, designed to standardize the structure and content of observational data and to enable efficient analyses that can produce reliable evidence. They are also thinking about a federated network, giving each team their autonomy, but with a minimal common data model that can be processed at local level using mutual computational and statistical methods. Annotation versus standardization could also be a solution to data harmonization, by using algorithms that can label individual data to help machines understand what exactly is in it and what is important.

The other key parameter to consider when merging data is reliability and how sensibly data should be integrated to minimize bias. New national approaches with robust designs for data collection are now emerging, like the UK COVID-19 REal-time Assessment of Community Transmission (REACT-1) programme [24], or the UK Office of National Statistics' studies. The work the University of Oxford is carrying out with ISARIC [20], integrating clinical data with epidemiological data, should make determining the representativeness of the information collected easier. Fiocruz also has in place a sensitivity and specificity strategy; when the system is picking up signals, they allow more sensitivity and not much specificity, but then they move quickly to enhance specificity by checking the signal plausibility. The validity of the outputs generated by integrated systems must also be checked. For instance, the WHO Global COVID-19 Incident Management Team has undertaken a preliminary retrospective evaluation of their alert system, using the data generated for weekly assessments between July 2021 and June 2022, to show that the framework generally provide timely and valid alerts for deteriorating situations, and provide recommendations for use of similar systems in future outbreaks.

Finally, ethical considerations must be considered in the process of integrating and sharing data. Detailed analysis can often involve potentially sensitive information, such as linking one or more of pathogen genetic sequences, demographic metadata, human genomics, clinical outcomes, and data from wearable devices. Dealing with large databases also requires security as it is important to keep a safe environment to protect privacy. All CIDACS [12]programs or proposals, as well as the whole data maintenance, are evaluated by an ethical board. They receive raw data directly from the ministries through a VPN, and they have designed their data lakes to be able to work both rapidly and safely. A data lake is a centralized repository designed to store, process, and secure large amounts of structured, semi-structured, and unstructured data. It can store data in its native format and process any variety of it, ignoring size limits. They manipulate their databases in safe rooms with high security, no access to the external environment, and no internet connections. The information that may be easier to re-identify is recoded in the safe room, where databases are linked and anonymized, in order to be ready for sharing during the subsequent analytical step. Similarly, the University of Oxford has built an open-source platform with relatively detailed but de-identified data. Algorithms identify entries in the platform that might pose privacy issues and guide the team to ascertain which fields should be aggregated or how they should obscure, for example, the geographic granularity of the information. This format makes it possible to share data openly, which is often a challenge for highly detailed clinical data.

### Creating opportunities for knowledge sharing on data integration

Public health intelligence is becoming analytically more sophisticated, combining both quantitative and qualitative data. The incumbent task is to make sure that decision makers are on the same page as domain experts working alongside with the analytic models of these data. They must be aware of the importance of the utilisation of epidemiological evidence in supporting their own political decisions, but they also need to be given tools to understand this evidence. In this context, JHU started providing assistance to different countries, giving priority to political processes that can be improved by technical support and analytical options. In their trainings, they teach the use of advanced analytical tools to generate public health scenarios, as well as how to translate classic data generation and synthesis into something useful for policymaking. They also provide technical support to different countries through the PAHO Health Information and Analysis Unit, and the backing of health situation rooms. The Health Situation Room concept is an opportunity to strengthen analysis and response within health systems using an innovative platform that allows governments, decision makers, and programme managers to integrate information, and easily analyse and view a selection of key indicators relevant to the country. The main value added of the solution is its capacity to merge data coming from multiple national data sources (DHIS, LMIS, community data, etc.) into one system [25]to offer a broader view of the factors involved in affecting the health status of diverse communities.

The dissemination and sharing of scientific knowledge is at the core of Fiocruz’s mission. Thus, AESOP [14] is going to be completely open access, comprising all the developments, information and sharing of solutions, so that anybody can learn from it and by using the tool. And with the collaboration of the Rockefeller Foundation’s Pandemic Prevention Initiative (PPI) [26], it will also be possible to have a larger reach, beyond Portuguese- speaking countries. The University of Oxford has also chosen to use an open-access platform that offers a stage for discussion and collaboration among scientists. On GitHub, most of the active debates happen through comments and GitHub issues. All changes to the database and any of the errors can be archived, so that people can engage and exchange knowledge, particularly on their local environments.

## Conclusion: moving forward

The ability to integrate multiple data streams has the potential to reshape the way that we conduct public health intelligence, but this will require a structured approach to data sharing, sustainability, and scalability, and the complexity of these systems may introduce challenges that could slow down epidemic preparedness.

A key challenge for data integration is the difficulty of making meaningful comparisons between countries. Differences in surveillance systems, levels of underreporting, and case definitions often limit the usefulness of direct comparisons of metrics such as case numbers or incidence. To enable more meaningful cross-country comparisons, efforts should focus on integrating information rather than just raw data, ensuring that contextual factors are taken into account in the analysis.After the COVID-19 pandemic, experts and politicians are looking at the global picture. In this context, coordination and unbiased data generation are critical, to ensure that recommendations and responses are relevant to all affected demographics and settings. However, reporting protocols may vary between public health agencies, as do their approaches to data collection and management, and running appropriate systems for integration still requires significant human resources. Ideally, countries should start producing data in formats that allow for information to be integrated using automated processes. To enable a more consistent response to comparable threats, guidelines should be developed to define early warning metrics in a way that ensures they have the same meaning across different contexts. While full harmonization may not always be feasible due to differences in surveillance capacities and data availability, establishing clear definitions and interpretation frameworks can improve comparability and strengthen early alert systems. More effort should be put into integrating contextual information, like testing protocols, vaccination rates, hospitalizations, health care capacity, or contact data. Ultimately, the goal should be to have a robust data pipeline that goes all the way from selecting the information needed to how it is collected, modelled, and communicated. Once digital tools are in place to analyse data very rapidly, more energy can be put into defining the type of data or the data points that must be considered in different phases of an epidemic, so that countries are reporting information of the same nature at the same time.

WHO could have a leading role in creating standards for metadata sharing, harmonization of epidemiological parameters, and defining labels for data quality. Labels should be developed not just for experts but also for policymakers, so that they can make decisions based on the reliability of the information they have. Moving forward, if this is done in collaboration with Ministries of Health and other key national stakeholders and operational partners, using a clear and transparent methodology and sufficiently accounting for the context in which the emergency is happening, wider sharing of these situational risk assessments may be possible. Clear communication of the potential benefits of standardizing alerts at a global level in terms of capacity building, advocacy, and resources prioritisation will be needed to encourage countries to participate in this approach.

Defining clear guidelines for upstream tasks such as data generation, metadata documentation and sharing, and methodological development as well as downstream implementation and communication would help make as much information as possible available for understanding future epidemics and pandemics. Moreover, a more robust approach to data generation and integration would facilitate a simpler and more transparent access to critical public health insights by multiple and diverse users. As routine use and sharing of large-scale epidemic datasets increase, there will also be a need for clear international ethical standards [27]. Moving forward, additional approaches to foster greater collaboration could be explored, such as building simulations with synthetic data, so that analysis specialists can still become familiar with available methods and digital tools for data integration without using real data.

The diverse and growing number of data sources relevant to public health presents a potential for conducting improved public health intelligence. However, to harness that potential, more robust approaches are needed so that these data can be integrated. During the COVID-19 pandemic, there were many innovative approaches to large-scale data integration. Building on these efforts will be important for detecting and preventing future pandemics and epidemics.

## Data Availability

Not applicable.

## Abbreviations

WHO: World Health Organization
EHR: Electronic Health Record
IPS: International Patient Summary
FHIR: Fast Healthcare Interoperability Resources
CDC: Centers for Disease Control and Prevention
JHU: Johns Hopkins University
OMOP: Observational Medical Outcomes Partnership
GHDx: Global Health Data Exchange
